# Immunomodulatory Activity of Polysaccharides Isolated from *Saussurea salicifolia* L. and *Saussurea frolovii* Ledeb

**DOI:** 10.3390/molecules28186655

**Published:** 2023-09-16

**Authors:** Igor A. Schepetkin, Marina G. Danilets, Anastasia A. Ligacheva, Evgenia S. Trofimova, Natalia S. Selivanova, Evgenii Yu. Sherstoboev, Sergei V. Krivoshchekov, Ekaterina I. Gulina, Konstantin S. Brazovskii, Liliya N. Kirpotina, Mark T. Quinn, Mikhail V. Belousov

**Affiliations:** 1 Department of Microbiology and Cell Biology, Montana State University, Bozeman, MT 59717, USA; igor@montana.edu (I.A.S.); kirpotina@hotmail.com (L.N.K.); 2 Goldberg Research Institute of Pharmacology and Regenerative Medicine, Tomsk NRMC, Tomsk 634050, Russia; m.danilets@mail.ru (M.G.D.); ligacheva_aa@pharmso.ru (A.A.L.); trofimova_es@pharmso.ru (E.S.T.); selivanova_ns@pharmso.ru (N.S.S.); sherstoboev_eu@pharmso.ru (E.Y.S.); 3 Pharmaceutical Faculty, Siberian State Medical University, Tomsk 634050, Russia; ksv_tsu@mail.ru (S.V.K.); e.gulina1@gmail.com (E.I.G.); bks_2005@mail.ru (K.S.B.); 4 Research School of Chemistry and Applied Biomedical Sciences, National Research Tomsk Polytechnic University, Tomsk 634050, Russia

**Keywords:** plant polysaccharide, *Saussurea*, macrophage, nitric oxide, cytokine, polymyxin B, xyloglucan

## Abstract

The genus *Saussurea* has been used in the preparation of therapies for a number of medical problems, yet not much is known about the therapeutic high-molecular-weight compounds present in extracts from these plants. Since polysaccharides are important in immune modulation, we investigated the chemical composition and immunomodulatory activity of *Saussurea salicifolia* L. and *Saussurea frolovii* Ledeb polysaccharides. Water-soluble polysaccharides from the aerial parts of these plants were extracted using water at pHs of 2 and 6 and subsequently precipitated in ethanol to obtain fractions SSP2 and SSP6 from *S. salicifolia* and fractions SSF2 and SSF6 from *S. frolovii*. The molecular weights of fractions SSP2, SSP6, SFP2, and SFP6 were estimated to be 143.7, 113.2, 75.3, and 64.3 kDa, respectively. The polysaccharides from *S. frolovii* contained xylose (67.1–71.7%) and glucose (28.3–32.9%), whereas the polysaccharides from *S. frolovii* contained xylose (63.1–76.7%), glucose (11.8–19.2%), galactose (4.7–8.3%), and rhamnose (6.8–9.4%). Fractions SSP2, SSP6, and SFP2 stimulated nitric oxide (NO) production by murine macrophages, and NO production induced by SSP2, SSP6, and SFP2 was not inhibited by polymyxin B treatment of the fractions, whereaspolymyxin B treatment diminished the effects of SFP6, suggesting that SFP6 could contain lipopolysaccharide (LPS). The LPS-free fractions SSP2, SSP6, and SFP2 had potent immunomodulatory activity, induced NO production, and activated transcription factors NF-κB/AP-1 in human monocytic THP-1 cells and cytokine production by human MonoMac-6 monocytic cells, including interleukin (IL)-1α, IL-1β, IL-6, granulocyte macrophage colony-stimulating factor (GM-CSF), interferon-γ, monocyte chemotactic protein 1 (MCP-1), and tumor necrosis factor (TNF). These data suggest that at least part of the beneficial therapeutic effects reported for water extracts of the *Saussurea* species are due to the modulation of leukocyte functions by polysaccharides.

## 1. Introduction

The genus *Saussurea* DC (family Asteraceae) is represented by about 500 species, which are widespread in Eurasia and North America and have long been used in folk medicine in the form of single- and multi-component herbal preparations. More than 200 compounds have been isolated and identified from *Saussurea* genus members, including phenylpropanoids, sesquiterpenoids, flavonoids, phytosterols, triterpenoids, lignans, coumarins, ceramides, and polysaccharides [1,2,3]. Extracts from *Saussurea* plants have been reported to exhibit anti-inflammatory, antioxidant, anti-cancer, anti-arthritic, and circulatory effects [4,5,6]. Among the putative therapeutic components of *Saussurea* species are polysaccharides. For example, polysaccharides from *S. involucrata* have been reported to have strong anti-melanogenic effects [7]. Likewise, polysaccharides from *S. laniceps* demonstrated anti-hepatitis B activity [8]. Lastly, *S. tridactyla* Sch. Bip.-derived polysaccharides were found to reduce cell apoptosis and protect cells from oxidative damage after UVB irradiation [9]. However, little is known about the immunomodulatory effects of *Saussurea* polysaccharides.

Botanical polysaccharides appear to modulate the immune system through their effects on macrophage function (reviewed in [10]). Macrophages and neutrophils represent the first lines of cellular defense in the body and are responsible for phagocytosing pathogens and killing tumor cells through the use of oxidative and nonoxidative killing mechanisms [11,12]. Macrophages can also serve as antigen-presenting cells and modulate acquired immune responses via antigen presentation to T cells [13]. Macrophages also have several other important roles, including tissue remodeling during embryogenesis, wound repair, and the clearance of apoptotic cells [14,15]. In efforts to enhance host defense against infection, recent research has focused on the development of therapeutics to enhance macrophage innate immune responses [16]. 

Since boiling in water is the most common mode for the preparation of herbal medicinal extracts, and the most common modes of administration are oral and local application, we hypothesized that *Saussurea* polysaccharides may have immunomodulatory properties and contribute to the therapeutic effects of extracts from this plant. To address this question, we fractionated water-soluble polysaccharides from *Saussurea* and evaluated their immunomodulatory activities in macrophage and monocytic cell assays. 

## 2. Results and Discussion

### 2.1. Partial Characterization of Saussurea Polysaccharides

The maximum yield was observed with a pH 6 extraction (Table 1); however, the highest hexose and uronic acid contents were found at a pH of 2. The content of the *O*-acetyl groups and protein was significantly increased with an increased extraction pH for *S. salicifolia* but did not change for *S. frolovii*. The significant difference in protein content for the fractions SSP2 and SSP6 could be explained by the presence of different functional groups in the protein molecules and their better solubility in neutral media. The samples obtained by acid extraction were characterized by the highest molecular weight (a significant difference was observed for the polysaccharide fractions from *S. salicifolia*). This may be due to the extraction of different polysaccharides, since the pH of the medium affects the solubility due to ionization of the molecules or the destruction of bonds with metal ions. An example of the homogeneity and average molecular weight analysis of the polysaccharide fractions is shown in Figure S1 (see Supplementary Material).

The monosaccharide composition of the *Saussurea* polysaccharides differed between the species and fractions (Table 2 and Supplementary Figure S2). The major monosaccharide, regardless of pH and plant species, was xylose. Glucose content was significantly higher in the polysaccharide fractions from *S. frolovii.* Galactose and rhamnose residues were found in the polysaccharides from *S. salicifolia* but were not present in the samples from *S. frolovii*. Arabinose and mannose were not found in any of the polysaccharide fractions. Thus, polysaccharides isolated from *S. frolovii* may be relatively pure xyloglucans.

IR spectroscopy established that there was a wide intense absorption band in the region of 3600–3200 cm^−1^ due to stretching vibrations of the O–H groups and absorption bands around 2932–2924 cm^−1^ in all samples, which is characteristic of the stretching and bending vibrations of C–H in carbohydrate rings. The spectra had similar absorption profiles over the entire range of wavelengths under study, differing only in the values of relative optical densities at wavelengths of 1725, 1601, 1160, and 1062 cm^−1^, which are characteristic of the stretching vibrations of carboxyl groups and explained by the differences in the relative uronic acid content of the fractions.

We next evaluated whether the *Saussurea* polysaccharides had helical structures using a Congo red assay, as Congo red can complex only with polysaccharides that have helical configurations [17,18]. When this complex is then treated with NaOH, the maximum absorption wavelength is red-shifted due to a weakening of the H-bond between OH-groups and the eventual destruction of the helix conformation. Therefore, this property can be used to detect whether the polysaccharide has a helical structure [17,18]. We found that in the spectra of *Saussurea* polysaccharides treated with Congo red, the maximum absorption wavelength was blue-shifted and was similar to that of pure Congo red, which decreased in tandem with increases in NaOH concentration. The one exception was fraction SFP2, for which the maximum absorption wavelength was not changed (Supplementary Figure S3). Thus, these data suggest that the conformation of *Saussurea* polysaccharides in solution is not triple-helical.

The sugar composition of polysaccharides from *S. involucrate* has recently been reported [19] and was mainly composed of arabinose, rhamnose, galactose, galacturonic acid, and glucose, with a molecular weight of 237.6 kDa. Pectin polysaccharide SLP-4 from *S. laniceps* was composed of mannose, rhamnose, galacturonic acid, glucose, galactose, xylose, and arabinose [8]. Thus, the sugar composition of polysaccharides from *S. salicifolia* has some similarity to those previously isolated from *S. involucrate* and *S. laniceps*, although fractions SFP2 and SFP6 did not contain rhamnose, and none of the fractions contained mannose and arabinose. 

### 2.2. Effects of Saussurea Polysaccharides on NO Production in Mouse Macrophages

**Figure 1:**
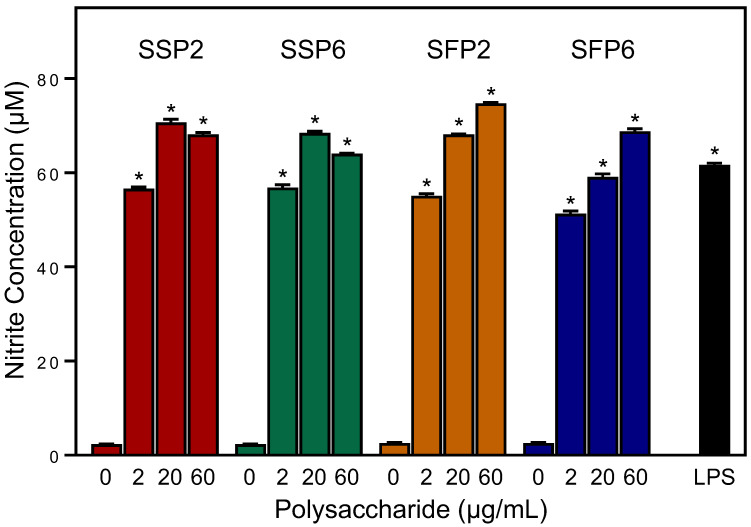
Effect of the *Saussurea* polysaccharides on macrophage NO production. Mouse macrophages were treated for 48 h with the indicated polysaccharide fractions, media alone (0; negative control), or 100 ng/mL LPS (positive control). NO production was quantified by measuring nitrite in the cell-free supernatants. The data are presented as the mean ± S.D. of triplicate samples from one experiment that is representative of two independent experiments. Statistically significant differences (* *p* < 0.01) between cells treated with media alone (0) and cells treated with SSP2, SSP6, SFP2, SFP6, or LPS are indicated.

To characterize effectiveness of the polysaccharide fractions in a primary macrophage cell model, we evaluated their effects in mouse peritoneal macrophages. While untreated macrophages (medium alone) did not produce NO, all the fractions (SSP2, SSP6, SFP2, and SFP6) stimulated significant levels of NO production at a concentration range of 2–60 μg/mL with similar activity as that induced by bacterial LPS (100 ng/mL) (Figure 1). 

**Figure 2:**
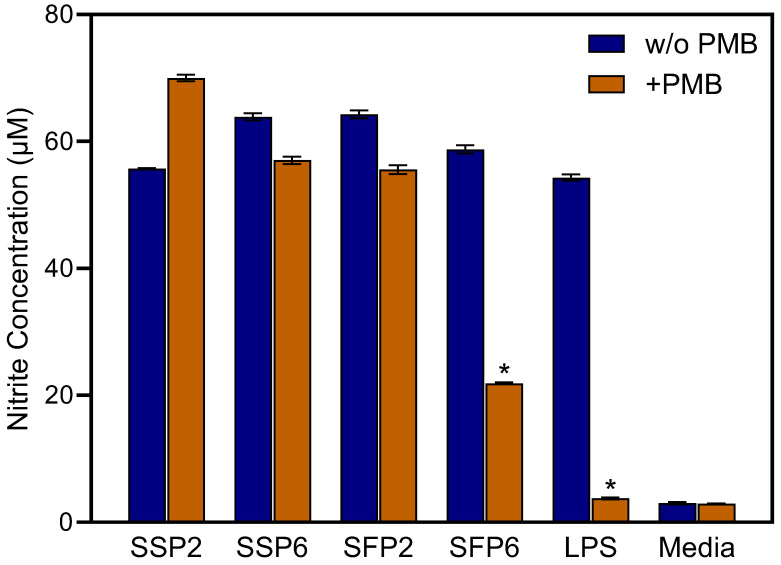
Effects of polymyxin B on NO production by polysaccharide-treated macrophages. Mouse macrophages pretreated with media (w/o PMB) or with 50 μg/mL polymyxin B (PMB) for 48 h were incubated with 20 μg/mL of the indicated polysaccharide fractions, media alone (negative control), or 100 ng/mL LPS (positive control). NO production was quantified by measuring nitrite in the cell-free supernatants. The data in each panel are presented as the mean ± S.D. of triplicate samples from one experiment that is representative of two independent experiments. Statistically significant differences (* *p* < 0.05) between samples treated with media and samples treated with polymyxin B are indicated.

To exclude possible effects of LPS or endotoxin contamination on the polysaccharide fractions, we evaluated activity of the polysaccharide samples in the presence of polymyxin B, which is an LPS inhibitor. As shown in Figure 2, NO production was not significantly affected in macrophages stimulated with SSP2, SSP6, and SFP2 in the presence of 50 μg/mL polymyxin B, indicating that these fractions were not contaminated with LPS. In contrast, NO production was significantly reduced by polymyxin B in macrophages treated with SFP6, as well as in the LPS control (Figure 2). Thus, fraction SFP6 was excluded from subsequent biological testing because of possible LPS contamination. Note that it is also possible that SFP6 contains structures similar to LPS, which would also be susceptible to polymyxin B binding and inhibition.

### 2.3. Effects of Saussurea Polysaccharides on Arginase Activity in Mouse Macrophages

Arginase I is a double-stranded manganese metalloenzyme that helps L-arginine break down into L-ornithine and urea [20]. Arginase activity is a marker of an M2 anti-inflammatory phenotype in macrophages [21]. The incubation of mouse macrophages for 48 h with polysaccharide fractions SSP2, SSP6, and SFP2 significantly decreased arginase activity compared to the negative control cells treated with media alone. The proinflammatory effect was comparable to that of LPS (100 ng/mL) for most of the polysaccharide samples, and only SFP2 was significantly lower (Table 3). 

### 2.4. Effects of Saussurea Polysaccharides on AP-1/NF-κB Transcriptional Activity

**Figure 3:**
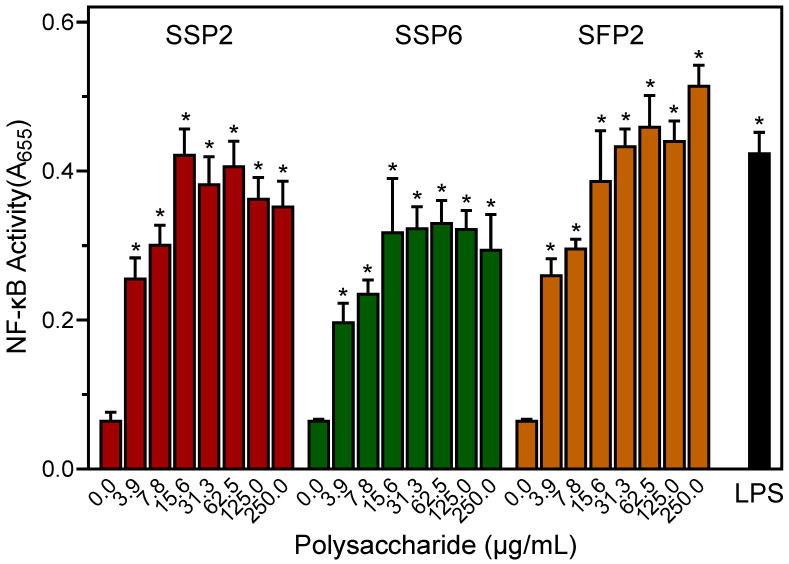
Effect of the *Saussurea* polysaccharides on AP-1/NF-κB activation. Human THP-1Blue monocytes (10^5^ cells/well) were incubated for 24 h with the indicated concentrations of polysaccharide, media alone (0; negative control), or 100 ng/mL LPS (positive control). Alkaline phosphatase activity was analyzed spectrophotometrically (absorbance at 655 nm) in the cell supernatants, as described. Values are the mean ± S.D. of triplicate samples from one experiment, which is representative of three independent experiments. Statistically significant differences (* *p* < 0.01) between cells treated with media alone (0) and cells treated with SSP2, SSP6, SFP2, or LPS are indicated.

To evaluate the activation of AP-1 and NF-κB transcription factors by *Saussurea* polysaccharides, we utilized a transcription factor-based bioassay in human THP-1Blue monocytic cells. As shown in Figure 3, all of the fractions dose-dependently stimulated AP-1/NF-κB transcriptional activity over a concentration range of 0 to 62.5 μg/mL. The maximal effects were comparable to those induced by 100 ng/mL of LPS (Figure 3). 

### 2.5. Effects of Saussurea Polysaccharides on Cytokine Production

**Figure 4:**
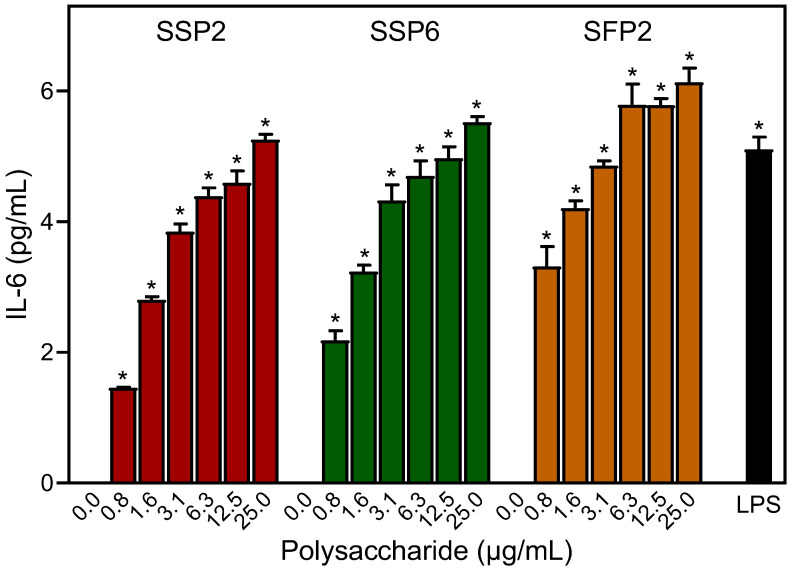
Effect of the *Saussurea* polysaccharides and LPS on IL-6 production. MonoMac-6 cells were pretreated with the indicated concentrations of polysaccharide, 100 ng/mL LPS, or media alone (0) for 24 h. Production of IL-6 in the supernatants was evaluated by ELISA. The data in each panel are presented as the mean ± S.D. of triplicate samples from one experiment that is representative of two independent experiments. Statistically significant differences (* *p* < 0.01) between cells treated with media alone (0) and cells treated with SSP2, SSP6, SFP2, or LPS are indicated.

To quantify the dose-dependent effects of the *Saussurea* polysaccharide fractions on cytokine production, the levels of monocyte interleukin-6 (IL-6) secretion were determined by ELISA in supernatants from polysaccharide-treated human MonoMac-6 monocytic cells. Incubation of MonoMac-6 cells with the polysaccharide fractions enhanced IL-6 production in a dose-dependent manner over a concentration range of 0.8 to 25 μg/mL (Figure 4), which is consistent with the NF-κB transcriptional activity data. 

**Figure 5:**
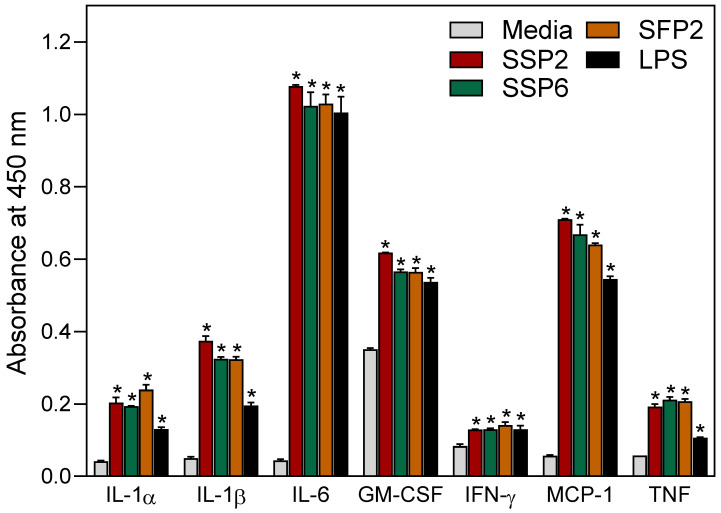
Effect of *Saussurea* polysaccharides on cytokine production by human MonoMac-6 cells. MonoMac-6 cells were incubated for 24 h with 20 μg/mL of the indicated polysaccharide fractions or 100 ng/mL of LPS (positive control), and production of cytokines in the supernatants was evaluated using a Multiplex Human Cytokine ELISA kit. The data are presented as mean ± SD of duplicate samples from one experiment that is representative of two independent experiments. Statistically significant differences (* *p* < 0.01) between cells treated with media alone and cells treated with the polysaccharide fractions or LPS are indicated.

As shown in Figure 1, Figure 2, Figure 3 and Figure 4, *Saussurea* polysaccharides were highly active in stimulating macrophage/monocyte functional responses at concentrations > 10 μg/mL. Thus, to determine whether the fractions induced the production of other pro-inflammatory mediators, conditioned media from polysaccharide-treated MonoMac-6 cells were analyzed using a Multiplex Cytokine ELISA array. Seven cytokines were consistently induced in the monocytic cells by 20 μg/mL of each polysaccharide fraction and compared to the control cells (treated with medium alone). These included interleukin (IL)-1α, IL-1β, IL-6, tumor necrosis factor (TNF), monocyte chemoattractant protein-1 (MCP-1), interferon-γ (IFN-γ), and granulocyte–macrophage colony-stimulating factor (GM-CSF) (Figure 5). We selected this concentration of the polysaccharides for the cytokine array analysis since the biological assays above generally showed maximal effects at or near this concentration. Additionally, the stimulatory effect was comparable to that induced by bacterial LPS (100 ng/mL).

The functional assays reported above suggest that the *Saussurea* polysaccharides were relatively non-toxic. Nevertheless, we evaluated the cytotoxic activity of our polysaccharide fractions to confirm that the biological results observed were not due to cytotoxicity. We found that none of the *Saussurea* polysaccharide fractions were cytotoxic, as indicated by the absence of any effect on macrophage viability when tested over a concentration range of 2−60 μg/mL. Thus, we confirmed that *Saussurea* polysaccharides are not cytotoxic for macrophages (see Supplementary Table S1).

Previously, the anti-inflammatory and immunomodulatory effects of *Saussurea* sp. extracts and bioactive molecules isolated from these plants have been described [22,23,24,25], whereas the immunomodulatory activities of high-molecular-weight polysaccharide fractions from these species are unknown. In the present work, we isolated two polysaccharide fractions from *S. salicifolia* (SSP2 and SSP6) and two fractions from *S. frolovii* (SFP2 and SFP6) and provided structural and pharmacological characterization. We found that the polysaccharides SSP2 and SSP6 from *S. salicifolia* had higher (113.2–143.7 kDa) molecular weights compared to SFP2 and SFP6 from *S. frolovii* (64.3–75.3 kDa). SSP2, SSP6, and SFP2 were free of endotoxin contamination and had potent immunomodulatory activity, as demonstrated by their ability to induce NO production in mouse macrophages and cytokines by monocytic MonoMac-6 cells.

The polysaccharides isolated from *S. frolovii* (SFP2 and SFP6) were mainly composed of glucose and xylose and seemed to be typical xyloglucans. Indeed, plant-derived xyloglucans have been previously reported to exhibit macrophage-mediated immunostimulatory activity [26,27]. Interestingly, we found that the extraction pH affected sugar composition of the fractions isolated from the same plant (see Table 2). Similarly, the effect of the extraction pH on structural properties was also reported for other plant polysaccharides (e.g., polysaccharides isolated from red pitaya (*Hylocereus polyrhizus*) stems [28]).

Although fraction SFP6 was excluded from biological testing because of possible endotoxin contamination, the other polysaccharides isolated from *S. frolovii* (SFP2) exhibited dose-dependent responses in all biological tests and had the most potent activity for stimulating NO production by murine macrophages as well as AP-1/NF-κB activation and IL-6 production by human monocytic cells. SFP2 contained the highest amount of glucose (32.9% vs. 11.8 and 19.2% in SSP2 and SSP6, respectively), but had the lowest molecular weight (75.3 kDa vs. 143.7 and 113.2 kDa for SSP2 and SSP6, respectively). Since LPS is a potent activator of monocyte and macrophages [29], we were not able to make any conclusions regarding the active components of SFP6. 

We show here that *Saussurea* polysaccharides were able to activate NF-κB/AP-1 in THP-1Blue human monocytes. This is an important finding and provides additional evidence that *Saussurea* polysaccharides exhibit monocyte/macrophage immunomodulatory effects, since NF-κB activation is important in the regulation of a number of the effector molecules involved in inflammation, including proinflammatory cytokines, chemokines, inflammatory enzymes, adhesion molecules, and receptors in innate immune cells [30]. Indeed, several other plant polysaccharide preparations have been shown to regulate NF-κB. For example, high-molecular-weight polysaccharides from *Aloe barbadensis* and *Opuntia polyacantha* have been reported to increase NF-κB expression and activity [31,32]. Likewise, polysaccharides from *Spirulina platensis*, *Aphanizomenon flos-aquae*, and *Chlorella pyrenoidosa* were reported to activate NF-κB, leading to increased cytokine message levels [33]. Thus, the ability of *Saussurea* polysaccharides to stimulate monocyte/macrophage IL-6 and NO production and activate NF-κB is consistent with the known immunomodulatory activity of various plant polysaccharides. 

The evaluation of arginase activity in mouse macrophages revealed that *Saussurea* polysaccharides could induce a phenotypic switch from M2 to M1 macrophages. This was also supported by our data demonstrating the activation of NO synthase and secretion of pro-inflammatory cytokines by the polysaccharides in macrophages/monocytes treated with the polysaccharide fractions SSP2, SSP6, and SFP2. A similar reversal of the macrophage phenotype was described after macrophage treatment with plant polysaccharides isolated from *Moringa oleifera* [34]. In future studies, it will be interesting to determine whether *Saussurea* polysaccharides can enhance the production of proinflammatory mediators through the activation of Toll-like receptor 4 (TLR4).

Note that the *Saussurea* polysaccharides had a relatively high protein content (from 6.5 to 33.9%). However, their biological activity was approximately the same in all tests, which indicates that the protein portion of these molecules was not the main component responsible for immunomodulatory activity. Due to complexity of the chemical structure of polysaccharides and the lack of accurate data on the relationship between their structure and activity, it is impossible to assess the exact contribution of the monosaccharide composition. However, there are data reported on some of the most important monosaccharide residues associated with macrophage stimulation. For example, Yin et al. [35] suggested that Ara, Man, Xyl, and Gal are the four most important monosaccharide components contributing to macrophage-stimulating activity. This conclusion was also supported by Wang et al. [36], who analyzed the monosaccharide composition and bioactivity of polysaccharides extracted from mushrooms. Likewise, we found that all active polysaccharide fractions had very high levels of Xyl, suggesting that the presence of this sugar may be important for monocyte/macrophage immunomodulatory activity. This conclusion is supported by the results observed with SFP2 treatment, as SFP2 contained only Xyl (~72%) and Glu (~28%). Considering the similar dose-dependent responses observed for SSP2, SSP6, and SFP2, we suggest that Xyl is likely the major structural contributor to the observed immunomodulatory activity since the Glu levels ranged from ~12 to 33% in these polysaccharide fractions and did not correlate well with the activities measured.

## 3. Materials and Methods

### 3.1. Plant Material

Plant material was collected from wild populations during the flowering period in July 2020 in the Republic of Khakassia, Russia (N 54.4669527, E 89.445091). Plant collection and botanical identification were performed by botanist Professor Margarita N. Shurupova from the Herbarium at Tomsk State University (Tomsk, Russia). The plant material was air-dried for 7–10 days at room temperature away from direct sunlight. 

### 3.2. Extraction and Fractionation of Saussurea Polysaccharides

The plant material was extracted with distilled H_2_O at pH 2 and 6 for 3 h at 60 °C and a volume ratio of 1/50 material/water [37,38]. The pH of the dispersion was adjusted using HCl or NaOH, as reported previously [39]. The volume of the extract was reduced by evaporation under vacuum. A four-fold volume of ethanol was added to each extract to precipitate the polysaccharides overnight at 4 °C. The precipitates were pelleted via centrifugation, dissolved in distilled H_2_O, and centrifuged at 2600× *g* for 15 min. After centrifugation, the polysaccharide solution was filtered through a 0.2 μm filter and lyophilized to obtain a dry substance.

### 3.3. Characterization of Saussurea Polysaccharide Fractions

The hexose content was determined using the phenol–H_2_SO_4_ method [40]. For the determination of uronic acid content, the samples were heat-treated in the presence of concentrated sulfuric acid. After cooling to room temperature, a 3,5-dimethylphenol solution was added, and 10–15 min later, the absorbance was read at 400 and 450 nm. The appropriate glucuronic acid standards were used to develop a standard curve [41]. The Lowry method was used to quantify protein content, with albumin as the protein standard [42]. The vibration of C–O in the *O*-acetyl groups was measured using the peak at 1260 cm^−1^ [43]. IR spectra were recorded on an analytical Fourier spectrometer FSM 2201 (LLC Infraspec, St. Petersburg, Russia) with KBr pellets.

The homogeneity and average molecular weight of the polysaccharide fractions were determined by high-performance size-exclusion chromatography (HP-SEC) using an Ultimate 3000 and Ultrahydrogel 250 column (7.8 mm × 300 mm, 250 Å (Waters, Milford, MA, USA)) eluted with water containing 10 mM sodium nitrate and 0.01% NaN_3_ at a flow rate of 0.5 mL/min at 30 °C. Peaks were detected using a refractive index detector RI-101 (Dionex, Thermo, Dreieich, Germany). The average molecular weights of the polysaccharide fractions were estimated through comparison with retention times of pullulan standards (Pulkitsa-10 Mp 342-708000 Da, PSS GmbH, Mainz, Germany). 

The monosaccharide composition was determined using gas chromatography. Then, 10 mg of polysaccharide was hydrolyzed using 2 M trifluoroacetic acid (TFA) for 4 h. The TFA was removed, and the samples were treated with trimethylchlorosilane (TMCS) and imidazole (3:1; total volume 100 μL) as derivatizing agents and pyridine (200 μL) as a solvent at 75 °C for 25 min [44]. TMCS-derivatized samples were twice extracted with hexane and analyzed using an Agilent 7890 (Agilent Technologies, Santa Clara, CA, USA) with an Agilent DB-5 GC column (30 m × 0.25 mm) and flame ionization detection (FID). The GC oven temperature was kept at 175 °C for 1 min, then increased to 250 °C at a rate of 3 °C/min.

The Congo red assay was used to determine the triple-helix structures of polysaccharides [17]. Briefly, 1 mL Congo red (80 μM/L) solution and 1 mL polysaccharide sample solution (1 mg/mL) were mixed, then NaOH solution and water were added to achieve a total volume of 4 mL while adjusting the NaOH final concentration from 0 to 0.5 M. After reaction for 10 min at room temperature, the solutions were analyzed at a wavelength of 400–600 nm with an SF-2000 spectrophotometer (OKB Spectr, St. Petersburg, Russia).

### 3.4. Cell Culture

Macrophages were isolated from C57BL/6 mice (age 8–10 weeks) obtained from the Department of Experimental Biological Models of E.D. Goldberg Institute of Pharmacology and Regenerative Medicine. We performed this research according to EU Directive 2010/63/EU concerning the protection of animals used for scientific purposes, and it was approved by the Animal Care and Use Committee of the Goldberg Research Institute of Pharmacology and Regenerative Medicine, Tomsk NRMC (Protocol No. 171052020 from 05.18.20).

Macrophages were isolated from peritoneal exudate using an EasySep™Biotin Positive Selection Kit and Anti-Mouse F4/80 Antibody (both from StemCell Technologies, Vancouver, BC, Canada). The macrophages were cultured in RPMI 1640 (Sigma-Aldrich, St. Louis, MO, USA) and supplemented with 10% (*v*/*v*) heat-inactivated, endotoxin-free fetal bovine serum (FBS) (Hyclone, GB), 20 mM HEPES (Sigma-Aldrich, St. Louis, MO, USA), 50 mM mercaptoethanol (Sigma-Aldrich, St. Louis, MO, USA), 2 mM L-glutamine (Sigma-Aldrich, St. Louis, MO, USA), and 50 μg/mL gentamycin.

THP-1Blue cells obtained from InvivoGen (San Diego, CA, USA) [45] and human monocyte–macrophage MonoMac-6 cells (Deutsche Sammlung von Mikroorganismen und Zellkulturen GmbH, Braunschweig, Germany) [46] were cultured at 37 °C in a humidified atmosphere containing 5% CO_2_, as reported previously.

### 3.5. Analysis of Macrophage Nitric Oxide (NO) Production

Macrophages (4 × 10^5^ cells/well) were plated in a final volume of 200 μL in 96-well flat-bottom tissue culture plates. The cells were incubated in control medium alone or in medium containing various concentrations of polysaccharide fractions or bacterial lipopolysaccharide (LPS; *Escherichia coli* K-235 serotype O111:B4 from Sigma-Aldrich, St. Louis, MO, USA) as a positive control. The macrophages were incubated at 37 °C and 5% CO_2_ for 48 h. After 48 h, 100 μL of the culture supernatants were removed and analyzed for nitrite with NaNO_2_ as the standard. Briefly, supernatants were mixed with an equal volume of Griess reagent (Sigma-Aldrich). After 20 min, absorbance was measured at 540 nm using a Titertek Multiskan^®^ MCC (Labsystems, Vantaa, Finland). 

### 3.6. Arginase Assay

The analysis of arginase activity was performed by measuring urea concentration in the cell lysate from 4 × 10^5^ cells using the Urea-450 colorimetric assay (Bio-LA-Test, Erba Lachema, Brno, Czech Republic). One unit of arginase enzymatic activity (E.U.) catalyzes the formation of 1 μM of urea per minute.

### 3.7. Analysis of AP-1/NF-κB Activation

The activation of AP-1/NF-κB was determined using an alkaline phosphatase reporter gene assay in THP1Blue cells, as reported previously [45].

### 3.8. Cytokine Analysis

A human IL-6 ELISA kit (BD Biosciences, San Jose, CA, USA) was used to measure MonoMac-6 IL-6 production, as reported previously [47]. A multiplex human cytokine ELISA kit from Anogen (Mississauga, ON, Canada) was also used to evaluate interleukin (IL)-1α, IL-1β, IL-6, the tumor necrosis factor (TNF), monocyte chemoattractant protein-1 (MCP-1), interferon-γ (IFN-γ), and the granulocyte–macrophage colony-stimulating factor (GM-CSF) in MonoMac-6 cell supernatants, as reported previously [47].

### 3.9. Cell Viability Assay 

The MTT (3-(4,5-dimethylthiazol-2-yl)-2,5-diphenyltetrazolium bromide) colorimetric assay was performed to determine cell viability. Macrophages were plated at 4 × 10^5^ cells/well in a 96-well plate and incubated for a 48 h with different concentrations of the plant polysaccharides, followed by the addition of 200 μg/mL of MTT reagent (Sigma-Aldrich, St. Louis, MO, USA). After a 4 h incubation, the supernatant was removed, and 100 μL of dimethyl sulfoxide was added to the cell pellet. The relative viable cell number was determined by reading the plates at a 490 nm wavelength using a Titertek Multiskan MCC (Labsystems, Vantaa, Finland).

### 3.10. Statistical Analysis

Statistical analysis was performed with Statistica 13.3 software. The compliance of the sample with a normal distribution was evaluated using Shapiro–Wilk’s W test, ANOVA, and Dunnett’s test. Values were considered statistically significant at *p* < 0.05.

## 4. Conclusions

The genus *Saussurea* has been used in the preparation of therapies for several medical problems, yet not much is known about the therapeutic compounds present in extracts from these plants. Since polysaccharides are important in immune modulation, we investigated the chemical composition and immunomodulatory activity of *Saussurea salicifolia* L. and *Saussurea frolovii* Ledeb polysaccharides. The data reported here demonstrate that *Saussurea* polysaccharides have potent monocyte/macrophage immunomodulatory properties, including activation of NO and cytokine production via the activation of the NF-κB/AP-1 transcriptional pathway. Thus, it appears that *Saussurea* polysaccharide may be able to enhance macrophage/monocyte host defense responses.

## Figures and Tables

**Table 1:** Chemical characteristics of *Saussurea* polysaccharide fractions extracted at pH 2 and pH 6.

Fraction	SSP2	SSP6	SFP2	SFP6
Yield (%)	1.13 ± 0.16	1.82 ± 0.10 *	1.50 ± 0.23	2.71 ± 0.11 *
Hexose (%)	44. 04 ± 3.53	33.88 ± 1.03 *	33.55 ± 5.77	23.98 ± 2.46
Uronic Acid (%)	7.71 ± 0.75	3.08 ± 0.42 *	4.76 ± 1.13	1.40 ± 0.27 *
*O*-acetyl group (μM/mL)	1.44 ± 0.05	2.30 ± 0.44 *	1.67 ± 0.24	1.61 ± 0.19
Protein (%)	6.51 ± 1.43	33.86 ± 6.78 *	13.65 ± 2.10	13.78 ± 1.54
M.W. (kDa)	143.66 ± 19.01	113.16 ± 16.64	75.29 ± 10.30	64.27 ± 6.55

* Significant differences (*p* < 0.05) vs. polysaccharide samples isolated at pH 2 from the same plant.

**Table 2:** Monosaccharide composition of *Saussurea* polysaccharide fractions.

Fraction	SSP2	SSP6	SFP2	SFP6
Glu	11.8 ± 0.2	19.2 ± 0.4 *	32.9 ± 0.6	28.3 ± 0.5 *
Gal	4.7 ± 0.1	8.3 ± 0.1 *	N.F.	N.F.
Xyl	76.7 ± 0.9	63.1 ± 0.8 *	67.1 ± 0.7	71.7 ± 0.7 *
Rha	6.8 ± 0.1	9.4 ± 0.1 *	N.F.	N.F.
Ara	N.F.	N.F.	N.F.	N.F.
Man	N.F.	N.F.	N.F.	N.F.

Abbreviations: Glu, glucose; Gal, galactose; Xyl, xylose; Rha, rhamnose; Ara, arabinose; Man, mannose. N.F., non-found. * Significant differences (*p* < 0.05) vs. polysaccharide samples isolated at pH 2 from the same plant.

**Table 3:** Effects of *Saussurea* polysaccharides on arginase activity.

Test Sample	Arginase Activity (E.U.)
SSP2 (20 μg/mL)	42.26 ± 0.43 *
SSP6 (20 μg/mL)	39.55 ± 0.64 *
SFP2 (20 μg/mL)	37.15 ± 0.64 *^#^
LPS (100 ng/mL)	45.67 ± 0.58 *
Control (media along)	53.94 ± 0.51

* Significant differences (*p* < 0.05) vs. negative (media alone) control. ^#^ Significant difference (*p* < 0.05) vs. LPS control.

## Data Availability

Not applicable.

## Supplementary Materials

The following supporting information can be downloaded at: https://www.mdpi.com/article/10.3390/molecules28186655/s1, Figure S1: High-performance size-exclusion chromatography (HP-SEC) analysis of homogeneity and average molecular weight of the polysaccharide fractions isolated from *Saussurea salicifolia* L. and *Saussurea frolovii* Ledeb. Polysaccharide fractions SSP2, SSP6, SFP2, and SFP6 were analyzed through HP-SEC and monitored with a refractive index detector, as described under Material and Methods. The arrows show peak retention times of the indicated pullulan standards used for calibration [P-200 (200 kDa), P-100 (11.3 kDa), P-50 (48.8 kDa), P-20 (23.0 kDa), and P-10 (9.9 kDa)]; Figure S2: Chromatograms of derivatized monosaccharides in the polysaccharide samples isolated from *S. salicifolia* (SSP2 and SSP6) and S. frolovii (SSF2 and SSF6) analyzed by gas chromatography/flame ionization detector (GC/FID). The retention times values of standards were 13.68, 14.38, 14.66, and 14.92 min for rhamnose, xylose, glucose, and galactose, respectively; Figure S3: Congo red analysis of the polysaccharide fractions; Table S1: Effect of *Saussurea* polysaccharide fractions on macrophage viability.
