# Supplementary material for: Immunomodulatory Activity of Polysaccharides Isolated from Saussurea salicifolia L. and Saussurea frolovii Ledeb
Source: Molecules. 2023 Sep 16;28(18):6655. doi: 10.3390/molecules28186655 (PMC10536955; doi:10.3390/molecules28186655)
Supplement: Supplementary file 1 [file molecules-28-06655-s001.zip › molecules-2545267-supplementary.pdf]

Supplementary Material

Immunomodulatory Activity of Polysaccharides Isolated from *Saussurea salicifolia* L. and *Saussurea frolovii* Ledeb.

Igor A. Schepetkin[1], Marina G. Danilets[2], Anastasia A. Ligacheva[2], Evgenia S. Trofimova[2,3], Natalia S. Selivanova[2], Evgenii Yu. Sherstoboev[2], Sergei V. Krivoshchekov[3], Ekaterina I. Gulina[3], Konstantin S. Brazovsky[3,4], Liliya N. Kirpotina[1], Mark T. Quinn[1], and Mikhail V. Belousov[3]

[1]Department of Microbiology and Cell Biology, Montana State University, Bozeman, MT 59717, USA

[2]Goldberg Research Institute of Pharmacology and Regenerative Medicine, Tomsk NRMC, Tomsk, 634050, Russia

[3]Siberian State Medical University, Tomsk, 634050, Russia

[4]National Research Tomsk Polytechnic University, Tomsk, 634050, Russia

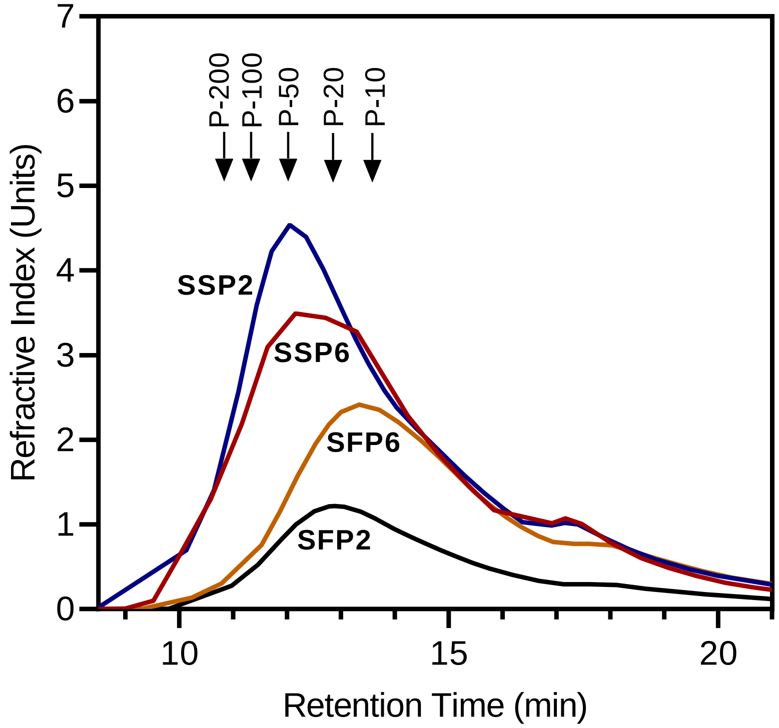

Figure S1. High performance size-exclusion chromatography (HP-SEC) analysis of homogeneity and average molecular weight of the polysaccharide fractions isolated from *Saussurea salicifolia* L. and *Saussurea frolovii* Ledeb. Polysaccharide fractions SSP2, SSP6, SFP2, and SFP6 were analyzed by HP-SEC and monitored with a refractive index detector, as described under Material and Methods. The arrows show peak retention times of the indicated pullulan standards used for calibration [P-200 (200 kDa), P-100 (11.3 kDa), P-50 (48.8 kDa), P-20 (23.0 kDa), and P-10 (9.9 kDa)].

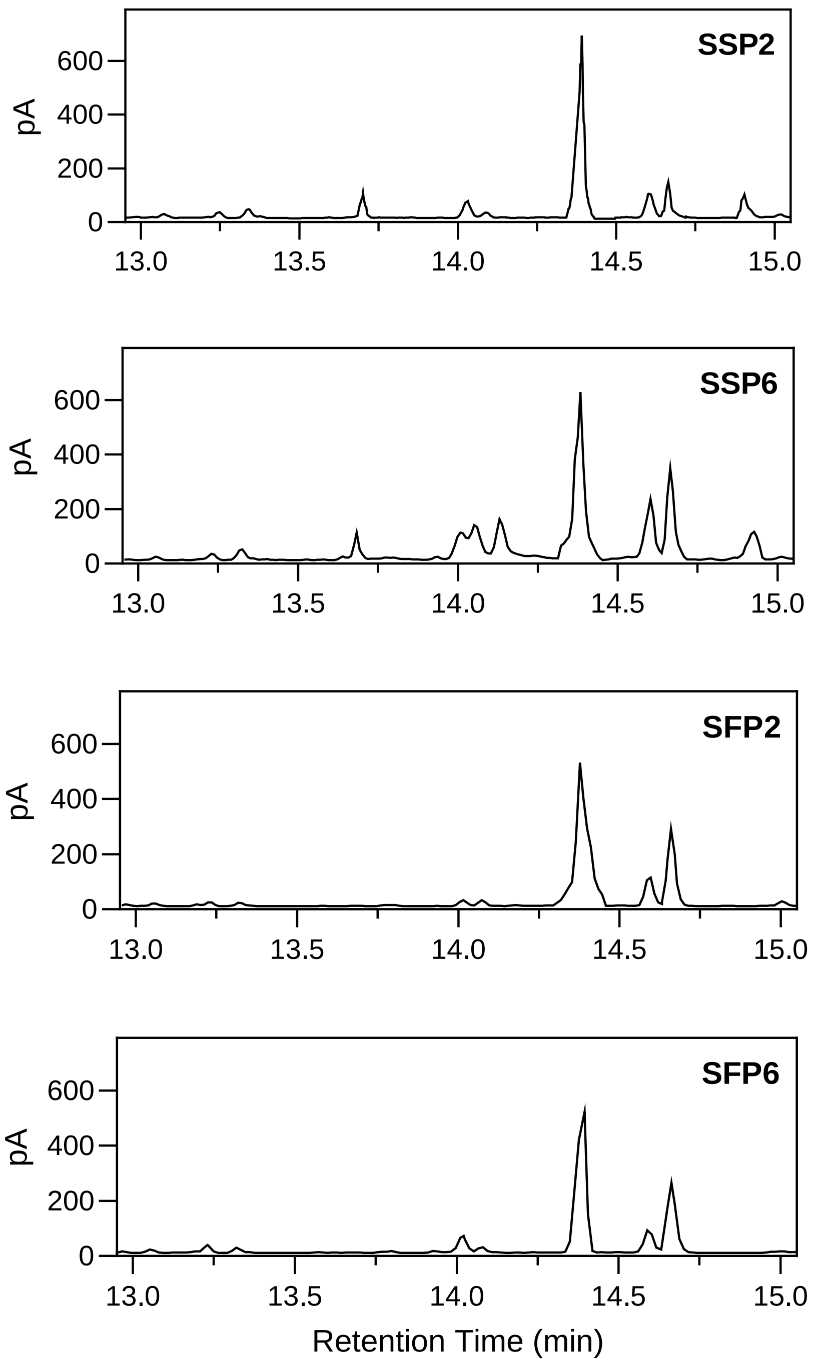

Figure S2. Chromatograms of derivatized monosaccharides in the polysaccharide samples isolated from S. salicifolia (SSP2 and SSP6) and S. frolovii (SSF2 and SSF6) analyzed by gas chromatography/flame ionization detector (GC/FID). The retention times values of standards were 13.68, 14.38, 14.66, and 14.92 min for rhamnose, xylose, glucose, and galactose, respectively.

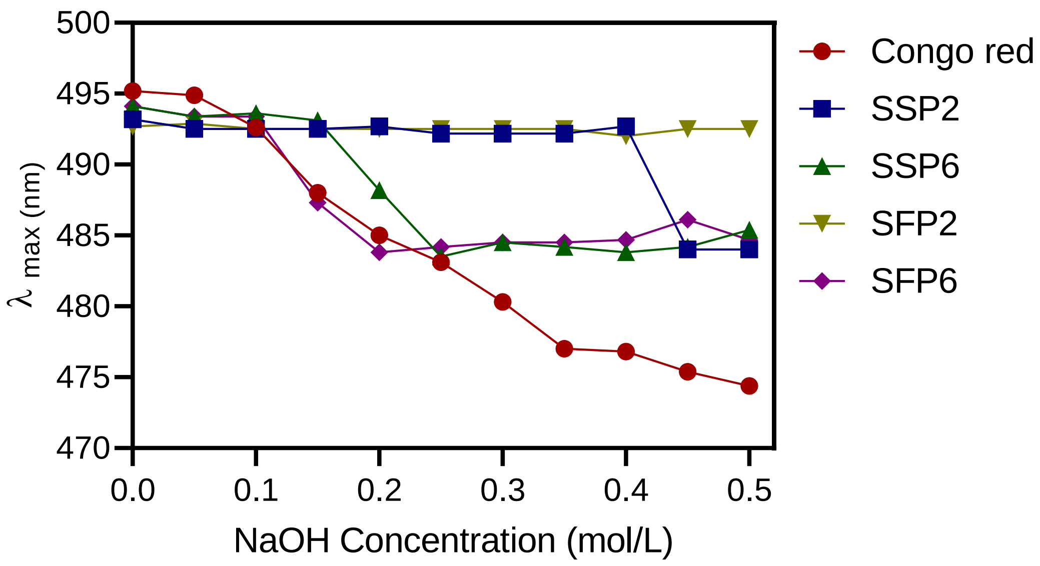

Figure S3. Congo red analysis of the polysaccharide fractions.

Table S1. Effect of *Saussurea* polysaccharide fractions on macrophage viability

	**SSP2**	**SSP6**	**SFP2**
**Concentration**	**Macrophage Viability (% from Media Control)**		
2 μg/mL	88.1 ± 1.0	95.7 ± 1.1	95.8 ± 1.9
20 μg/mL	93.9 ± 1.5	97.7 ± 1.8	87.7 ± 0.4
60 μg/mL	87.1 ± 1.6	96.9 ± 0.5	85.8 ± 0.2
